# Tricaprylin‐based drug crystalline suspension for intramuscular long‐acting delivery of entecavir with alleviated local inflammation

**DOI:** 10.1002/btm2.10649

**Published:** 2024-01-29

**Authors:** Min Young Jeong, Myoung Jin Ho, Joon Soo Park, Hoetaek Jeong, Jin Hee Kim, Yong Jin Jang, Doe Myung Shin, In Gyu Yang, Hye Rim Kim, Woo Heon Song, Sangkil Lee, Seh Hyon Song, Yong Seok Choi, Young Taek Han, Myung Joo Kang

**Affiliations:** ^1^ College of Pharmacy, Dankook University Cheonan Chungnam Republic of Korea; ^2^ College of Pharmacy, Chung‐Ang University Seoul Republic of Korea; ^3^ College of Pharmacy, Kyungsung University Busan Republic of Korea

**Keywords:** delivery vehicle, drug microparticle, drug particle aggregation, intramuscular injection, local inflammation, oil suspension, systemic exposure, Tricaprylin

## Abstract

In order to ensure prolonged pharmacokinetic profile along with local tolerability at the injection site, tricaprylin‐based drug crystalline suspension (TS) was designed and its local distribution, pharmacokinetics, and inflammatory response, were evaluated with conventional aqueous suspension (AS). As model drug particles, entecavir 3‐palmitate (EV‐P), an ester lipidic prodrug for entecavir (EV), was employed. The EV‐P‐loaded TS was prepared by ultra‐sonication method. Prepared TS and conventional AS exhibited comparable morphology (rod or rectangular), median diameter (2.7 and 2.6 μm), crystallinity (melting point of 160–165°C), and in vitro dissolution profile. However, in vivo performances of drug microparticles were markedly different, depending on delivery vehicle. At AS‐injected site, drug aggregates of up to 500 μm were formed upon intramuscular injection, and were surrounded with inflammatory cells and fibroblastic bands. In contrast, no distinct particle aggregation and adjacent granulation was observed at TS‐injected site, with >4 weeks remaining of the oily vehicle in micro‐computed tomographic observation. Surprisingly, TS exhibited markedly alleviated local inflammation compared to AS, endowing markedly lessened necrosis, fibrosis thickness, inflammatory area, and macrophage infiltration. The higher initial systemic exposure was observed with TS compared to AS, but TS provided prolonged delivery of EV for 3 weeks. Therefore, we suggest that the novel TS system can be a promising tool in designing parenteral long‐acting delivery, with improved local tolerability.

**Translational Impact Statement**

To the best of our knowledge, we firstly established that the type of delivery vehicle has considerably influenced on the pharmacokinetic profile and local tolerability of injectable drug suspension. The novel tricaprylin‐based suspension system provided prolonged pharmacokinetic profile with lessened local inflammatory response at the injection site, compared to conventional aqueous suspension. We suggest that the neutral oil‐based crystalline system could be an alternative in designing intramuscular long‐acting delivery of the water‐insoluble drug particles for clinical application, with alleviate local inflammation.

## 1 INTRODUCTION

Parenteral long‐acting (LA) delivery systems have been increasingly exploited to provide long‐term (i.e., weeks to months) therapeutic effect following a single injection, with the aim of improving treatment outcomes.^1^, ^2^ Several pharmaceutical approaches have been explored in designing injectable LA systems of chemicals, including polymeric microparticles, liposomes, in situ gelling/implant systems, and drug crystalline suspensions.^3^, ^4^, ^5^, ^6^, ^7^ Injectable drug suspensions generally consist of nano‐ or micrometer sized (pro)drug particles suspended in aqueous vehicles with minimal use of steric or electrostatic stabilizers (surfactants or hydrophilic polymers).^8^, ^9^ This carrier‐free LA system allows high drug‐loading, effective particle size control, and long‐term stability with well‐established fabrication process, leading to its successful commercialization including DepoMedrol® (methylprednisolone acetate), Depo‐Provera® (medroxyprogesterone acetate), Kenalog® (triamcinolone acetonide), Invega Sustenna® (paliperidone palmitate), Abilify Maintena® (aripiprazole), Aristada® (Aripiprazole Lauroxil), and Cabenuva® (cabotegravir and rilpivirine).^2^, ^10^ Following intramuscular (IM) injection of drug suspensions, the low solubility of hydrophobic (pro)drug particles at the injection site principally affords protracted dissolution and absorption into the bloodstream, providing prolonged pharmacokinetic profile.^8^, ^11^ In vivo drug dissolution and subsequent absorption process at injection site can be influenced by aggregation and/or spreading behavior of the drug nano or microparticles, protein adsorption on the particular surface, enzymatic (pro)drug degradation, and lymphatic transportation.^6^, ^12^, ^13^, ^14^, ^15^, ^16^ Moreover, recent investigations revealed that in vivo drug absorption of drug crystalline suspensions is affected by elicited injection site reaction against drug particles recognized as exogeneous materials at injection site.^10^ The foreign‐body reaction against drug particles is mainly initiated by the action of monocytes and macrophages upon adherence to the material surface. Afterwards, fibroblasts and vascular endothelial cells at the injection or implant site begin to form granule, isolating the drug particles or implants.^17^, ^18^

Several studies have shown that the in vivo behavior of injectable suspensions is affected by physiological factors (injection site, depth, or muscular activity), intrinsic drug properties (solubility, biocompatibility, or cytotoxicity), the physical properties of drug particles, and the nature of delivery vehicle.^2^, ^19^, ^20^, ^21^ The natures of the delivery vehicle, including viscosity, hydrophilicity/lipophilicity, and pH, affect the local distribution of the drug particles over the injection site, diffusion of the drug in the vehicle, and thus the extent and rate of drug absorption.^22^, ^23^, ^24^, ^25^ The dispersion of drug suspension along muscle fibrils varies depending on the viscosity and the affinity of the vehicle with interstitial fluid and tissues, which influences the interfacial area between the drug particle and aqueous media.^12^, ^22^ In previous studies, oily vehicles including vegetable oils have been employed to formulate drug suspension system of water‐soluble drugs.^12^ Oily vehicles typically have retarded disappearance rate at injection sites compared with aqueous vehicles.^26^, ^27^ The oily vehicle retarded the dissolution of the water‐soluble drugs by hindering the contact with interstitial fluid, enzymes, and ions, resulting in the drug sustained release.^28^ Moreover, several oily vehicles including medium‐chain triglycerides (MCTs) have reported to possess anti‐inflammatory effect; in a previous study, murine macrophages (RAW 264.7) incubated with MCT showed markedly suppressed inflammatory cytokine secretion in lipopolysaccharide‐induced conditions, downregulating the production of interleukin (IL)‐6 and tumor necrosis factor (TNF)‐α secretion.^29^ Therefore, oily vehicles might have a greater influence on biological responses, such as local inflammation against injected drug particles or implants with its extended retention time. However, to the best of our knowledge, studies are yet to formulate oily vehicle‐based drug crystalline suspension (OS) for poorly water‐soluble compound and to comprehensively evaluate the effect of delivery vehicle types on drug dispersion and/or aggregation, local inflammatory response, and pharmacokinetic behavior of injectable suspensions.

Therefore, the principal objectives of this study were to design OS for the water‐insoluble drug candidate and to compare in vivo behavior with conventional aqueous suspension (AS), along with drug particles aggregation and the disappearance of vehicle from the injection site. An ester prodrug for entecavir (EV), 2‐amino‐1,9‐dihydro‐9‐[(1*S*,3*R*,4*S*)‐4‐hydroxy‐3‐(hexadecanoyloxymethyl)‐2‐methylenecyclopentyl]‐6*H*‐purin‐6‐one (EV‐P) was employed as model drug. This lipidic prodrug is highly lipophilic (calculated log *P* value of 5.73) with extremely low aqueous solubility (approximately 1.1 μg/mL in phosphate‐buffered saline [PBS], pH 7.4).^30^ A previous study showed that subcutaneous (SC) or IM injection of AS of EV‐P provided prolonged pharmacokinetic profile of EV for over 2 weeks in beagle dogs.^30^ Herein, injectable OS of EV‐P was prepared using ultrasonication methods after dispersibility and solubility screening in injectable oils. Both suspensions were physicochemically characterized by morphology, crystal size, drug crystallinity, viscosity, and in vitro dissolution profile. Drug concentration–time profiles in plasma and drug remaining at the injection site were evaluated following IM single injection in rats. The disappearance of the vehicles from the femoral tissue of rats was further evaluated using micro‐computed tomography (micro‐CT). Moreover, the differences in the aggregation behavior of EV‐P particles and associated inflammatory responses depending on delivery vehicles were electron‐microscopically and histologically evaluated, and their relationship with the pharmacokinetic pattern was investigated.

## 2 RESULTS AND DISCUSSION

### 2.1 Selection of oily vehicle to formulate OS of EV‐P

Recent advance in particle engineering technology enables the fine‐tuning of particle size, size distribution, shape, density, outer or inner structure, and/or powder properties, and those effects on in vivo performance have been investigated.^31^, ^32^ For instance, the particle size of injectable drug suspensions directly affects the dissolution and absorption rate, as established by Noyes–Whitney equation.^6^, ^10^ Moreover, drug crystal size affects the inflammatory area, infiltration density of inflammatory cells, and thickness of fibroblastic bands around drug particles, highly varying the degree of engulfment and association with macrophages and subsequent cytokine secretion.^6^, ^18^ Moreover, the drug nanocrystals can be absorbed by macrophages or be drained directly into thoracic lymphatic vessels and subsequently absorbed into systemic circulation.^33^, ^34^ However, to the best of our knowledge, the effect of delivery vehicle on the in vivo performance of drug suspension has not investigated yet. Designing both OS and AS for the same drug compound is challenging because of the difficulty in homogeneously dispersing drug particles in both oily and aqueous vehicles, respectively. In particular, it is quite challenging to formulate OS system for lipophilic compounds, because of high solubility in oily vehicle with difficulty in control of interfacial phenomenon. However, we assure that a straightforward comparison of the effects of different vehicles on the local response and pharmacokinetic profile of the same drug might provide valuable insights for the rational design of injectable suspensions.

The dispersibility and solubility of the lipidic compound in different injectable oils were screened to formulate OS system of EV‐P (Table 1). Oleic acid (5.9 mg/mL), castor oil (2.1 mg/mL), and peanut oil (1.9 mg/mL) showed high drug solubility (>1 mg/mL). Additionally, the lipidic prodrug was soluble in soybean oil (0.80 mg/mL) and cotton seed oil (0.98 mg/mL) containing several saturated or unsaturated fatty acids. In contrast, the prodrug was poorly soluble in tricaprylin (0.03 mg/mL) and Labrafac PG (0.11 mg/mL) possessing triester of glycerin or caprylic acid structure, respectively, despite the high lipophilicity of EV‐P (log *P* value of 5.73) and oily vehicles (log *P* values of tricaprylin and Labrafac PG are 8.25 and 3.97).^35^, ^36^ Compared with the other oily vehicles, less than 1.0% (w/v) of EV‐P was dissolved in OS designed using tricaprylin and Labrafac (EV‐P concentration of 9.0 mg/mL) because of their low solubility. Moreover, the drug particles were evenly dispersed in tricaprylin and Labrafac PG, with no aggregation. Therefore, tricaprylin was selected as injectable oily vehicle for preparing the OS system (named TS) because of its lower solubility and higher dispersibility for drug particles compared with Labrafac PG.

**TABLE 1:** Solubility and dispersibility of EV‐P drug particles in different oily vehicles.

Vehicle	Solubility (mg/mL)^a^	Dispersibility^b^
Castor oil	2.14 ± 0.07	Aggregated
Peanut oil	1.95 ± 0.18	Aggregated
Soybean oil	0.80 ± 0.13	Aggregated
Tricaprylin	0.03 ± 0.01	Re‐dispersible
Oleic acid	5.90 ± 0.05	Re‐dispersible
Labrafac PG	0.11 ± 0.01	Aggregated
Miglyol 812	0.62 ± 0.12	Aggregated
Cotton seed oil	0.98 ± 0.57	Aggregated

Abbreviation: EV‐P, entecavir 3‐palmitate.

^a^ Data are expressed as mean ± SD (*n* = 3).

^b^ Assessed after 3 days storage at 25°C. The concentration of EV‐P in the vehicles was set to 9.0 mg/mL.

### 2.2 Preparation and physicochemical characteristics of EV‐P TS and AS

EV‐P‐loaded TS and AS were prepared using a sonication‐assisted top‐down method and an anti‐solvent crystallization technique, respectively, and their morphological and physical characteristics were evaluated. There are several approaches to fabricate drug suspensions generally classified as top‐down, bottom‐up, and combination approaches.^37^, ^38^ In top‐down methods, the coarse drug powders were crushed using high energy approaches such as wet milling and high‐pressure homogenization. In bottom‐up techniques, small and uniform drug particles dispersed in aqueous media are obtained after precipitation from a supersaturated drug solution. During TS preparation, ultrasonic treatment pulverized the large clusters of the lipidic powder into small microcrystals, breaking the intermolecular interactions, such as van der Waals forces, between lipidic moieties. However, AS was prepared using anti‐solvent precipitation method because of technical difficulties using the top‐down method. The water‐insoluble prodrug dissolved in ethanol was promptly precipitated upon contact with the aqueous vehicle to form fine aggregates with steric stabilizers, including polysorbate 20 and PEG 4000.

**FIGURE 1:**
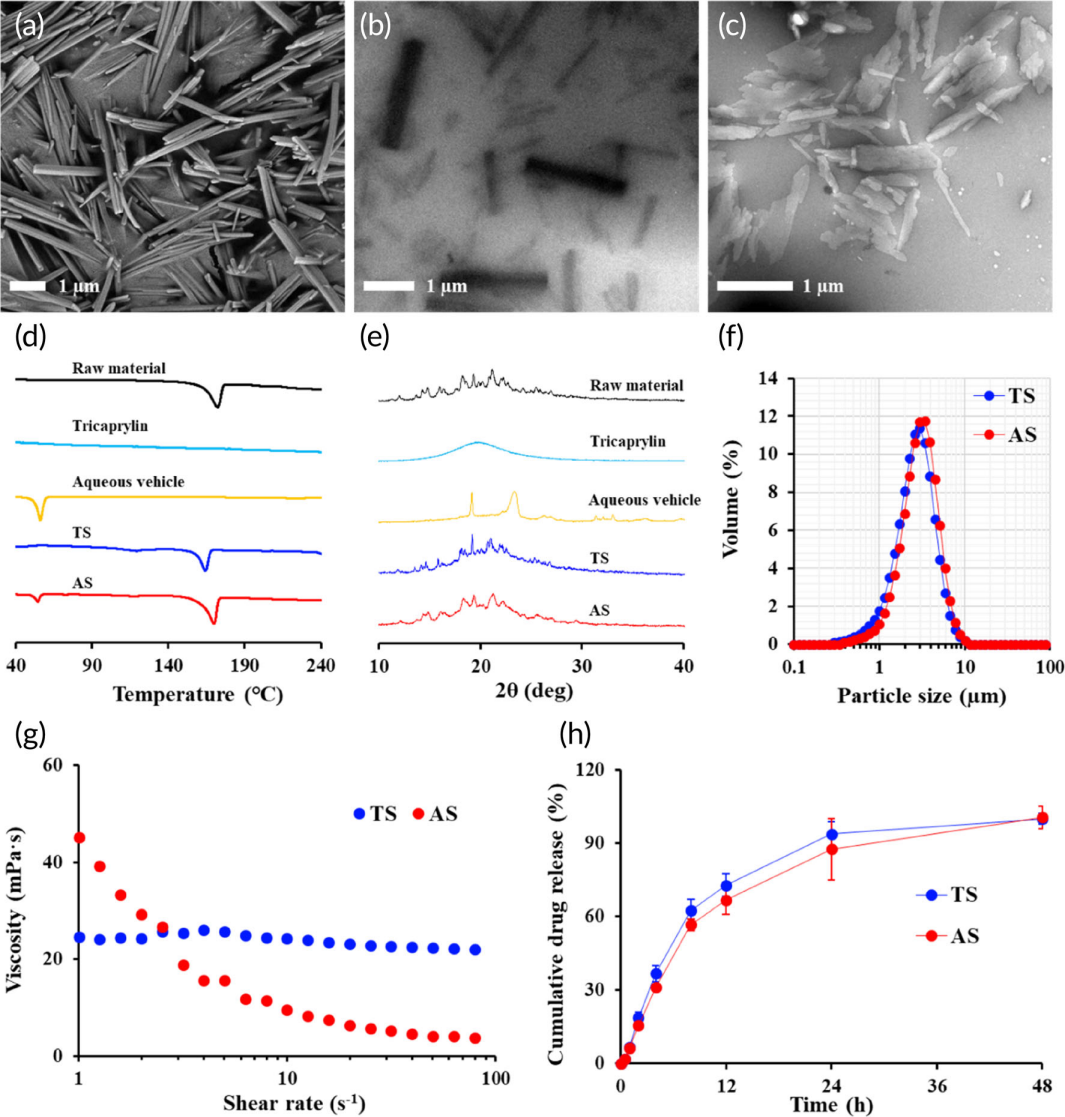
Morphology, physical characteristics, and in vitro dissolution profile of EV‐P TS and AS. (a) Scanning electron microscope (SEM) image of EV‐P powder. Transmission electron microscopic (TEM) images of drug particles in (b) TS and (c) AS, respectively. (d) Representative differential scanning calorimetry and (e) x‐ray diffraction patterns of the raw material, tricaprylin, aqueous vehicle, and EV‐P suspensions. (f) Size distribution of EV‐P particles suspended in aqueous and oily vehicles. (g) Representative viscosity curve of drug suspensions in the shearing rate of 1–100 s^−1^. (h) In vitro dissolution profile of EV‐P microparticles from TS and AS loaded in dialysis bag under sink condition. The sink condition was guaranteed by adding isopropyl alcohol to phosphate buffer (pH 7.4) (50:50, v/v%). AS, aqueous suspension; EV‐P, entecavir 3‐palmitate; TS, tricaprylin‐based drug crystalline suspension.

Transmission electron microscopy (TEM) showed that EV‐P crystals dispersed in the tricaprylin exhibited a rod‐shaped crystalline form (Figure 1b), as observed in the drug powder (Figure 1a). The lengths of the major axis and minor axis of the drug crystals in TS were estimated to be approximately 0.5–3.0 μm and 0.1–0.3 μm, respectively, with median diameter (*d*_50_) of 2.7 μm (Figure 1f, Table 2). The shape of drug crystals in AS, formed through drug aggregation in aqueous vehicle, was also rod or rectangular, with irregular and rough surface properties compared to the crystals in TS (Figure 1c). Despite the differences in the shape and surface properties of drug crystals, the *d*_50_ of AS was 2.6 μm (Table 2), which was similar to that of TS. The size distribution of EV‐P in both suspensions was homogeneous, with SPAN values lesser than 1.4 (Table 2). In the present study, the diameter of the particles in the suspensions was adjusted to less than 3.0 μm, which is the particle size commonly employed in parenteral suspensions. Suspensions with smaller particles provide higher drug loading with lower viscosity, but may cause initial burst release and shorten the duration of systemic exposure. In contrast, particle size greater than 10 μm increased the viscosity of the injectable suspension considerably, thus reducing the ease of administering the suspension (data not shown). Generally, the mean particle size of aripiprazole (Abilify maintena®, Otsuka Pharmaceutical Inc., Tokyo, Japan), triamcinolone acetonide (Kenalog‐40®, Bristol–Myers Squibb, NY, USA), paliperidone palmitate (Invega sustenna®, Janssen, NJ, USA), and olanzapine suspensions (Zyprexa Relprevv®, Eli Lilly, Indianapolis, IN, USA) are reported to be approximately 1–10 μm, 1–10 μm, < 3 μm, and 1–13 μm, respectively.^39^, ^40^, ^41^, ^42^ In the present study, drug concentration in both suspensions was adjusted to 8.5–9.3 mg/mL as EV‐P (4.6–5.0 mg/mL as EV), and more than 99.5% of the lipidic prodrug remained as solid‐state drug particles in both oily and aqueous vehicles (Table 2). Differential scanning calorimetry (DSC) analysis revealed that there was no significant difference in the thermal behaviors of drug microcrystals dispersed in TS and AS, with endothermic peaks ranging from 165 to 170°C (Figure 1d). The identical melting point, a crystal lattice energy that is required to break down the molecule from the crystalline structure, denotes that the crystallinity of drug particles dispersed in TS and AS are analogous, despite the partial variations in the shape of drug crystals. The endodermic peak observed at about 56°C in AS was confirmed to be originated from the desiccated aqueous vehicle composed with PEG 4000, Tween 80, and phosphate salt. The additional XRD analysis revealed that diffraction patterns of EV‐P powder, and microcrystals dispersed in AS and TS were analogous (Figure 1e), exhibiting distinctive peaks at 18.2°, 19.3°, and 21.2°. Thus, we concluded that there was no difference in crystalline form between AS and TS, with no marked alternation in crystalline form during the preparation process.

**TABLE 2:** Physicochemical characteristics of EV‐P TS and AS.

Parameters	TS	AS
EV‐P conc. (mg/mL)^a^		
Suspended (mg/mL)	9.32 ± 0.09	8.77 ± 0.41
Dissolved (mg/mL)	0.03 ± 0.00	0.04 ± 0.00
Particle size		
*d*_0.1_ (μm)^a^, ^b^	1.35 ± 0.19	1.36 ± 0.08
*d*_0.5_ (μm)^a^, ^c^	2.71 ± 0.26	2.64 ± 0.22
*d*_0.9_ (μm)^a^, ^d^	4.96 ± 0.40	4.69 ± 0.21
Size distribution (SPAN)^a^, ^e^	1.33 ± 0.15	1.27 ± 0.08
Zeta potential (mV)^a^	n.d.^g^	−6.2 ± 1.7
Viscosity (cP)^a^, ^f^	45.2	24.5

Abbreviations: AS, aqueous suspension; EV‐P, entecavir 3‐palmitate; TS, tricaprylin‐based drug crystalline suspension.

^a^ Data are expressed as mean ± SD (*n* = 3).

^b^ Particle sizes below 10% (*d*_0.1_) of the total particles. The crystal size was calculated based on the volume weighted diameter.

^c^ Particle sizes below 50% (*d*_0.5_) of the total particles. The crystal size was calculated based on the volume weighted diameter.

^d^ Particle sizes below 90% (*d*_0.9_) of the total particles. The crystal size was calculated based on the volume weighted diameter.

^e^ Calculated by dividing the difference between *d*_0.9_ and *d*_0.1_ by *d*_0.5_.

^f^ Determined at 25°C with shearing rate of 1 cm^−1^.

^g^ Not determined.

Figure 1g shows the apparent viscosity curves of TS and AS, which were determined using a rotational shear rheometer. The viscosities of AS and TS under storage conditions (shearing rate of 1 s^−1^) were 45.2 and 24.5 centipoise (cP), respectively. AS exhibited a pseudoplastic flow pattern, with a decrease in viscosity with increase in shear rate, whereas TS showed even apparent viscosity in the range of 1–100 cm^−1^. Both formulations exhibited apparent viscosity below 50 cP (Figure 1g, Table 2), which allowed the formulations to sufficiently pass‐through syringes as small as 31 G (data not shown).

### 2.3 In vitro dissolution profile of EV‐P TS and AS

The in vitro dissolution profiles of EV‐P TS and AS were comparatively evaluated using the dialysis bag diffusion model. The dialysis membrane prevents the unrestricted movement of drug particles in dissolution media, mimicking the conditions at injection sites, restricting the movement of the injected drug particle and allowing partition into tissue and/or blood stream. To provide sink conditions for EV‐P and miscibility for oily vehicles, a mixture of isopropyl alcohol and distilled water (5:5, v/v) was employed as the dissolution medium. The solubility of EV‐P in the phosphate buffer (pH 7.4) was 1.1 μg/mL, whereas the solubility in the isopropyl alcohol and distilled water (5:5, v/v) mixture was over 100 μg/mL. Generally, dissolution media containing co‐solvents, such as isopropyl alcohol, have been employed to provide sink conditions in dissolution experiments for poor water‐soluble compounds.^43^, ^44^, ^45^

Figure 1h shows the release profiles of the lipidic prodrug from TS and AS under sink conditions at 37°C. In both suspensions, the prodrug was gradually dissolved over the experimental period, with about 60% of the drug released after 8 h and over 87% after 24 h. There was no noticeable difference in the dissolution profile of TS and AS systems, with similar median diameters, and thus comparable effective surface areas. The similarity of the dissolution profiles of AS and TS was further compared by fitting into Equation (1).^46^

(1) $$f_{2}=50∙\log\left\{{\left[1+\frac{1}{n}\sum_{t=1}^{n}{\left({\mathrm{AS}}_{t}-{\mathrm{TS}}_{t}\right)}^{2}\right]}^{-0.5}\times 100\right\},$$

where *f*_2_ is the similarity factor, *n* is the number of time points, and TS_*t*_ and AS_*t*_ are the dissolution values of TS and AS at time *t*, respectively. The two dissolution profiles are considered to be similar when the *f*_2_ value range between 50 and 100.^46^ The *f*_2_ value was calculated to be 68.8, suggesting that there was no significant difference in dissolution patterns of the two formulations.

### 2.4 In vivo pharmacokinetic profile following IM injection of EV‐P TS or AS

**FIGURE 2:**
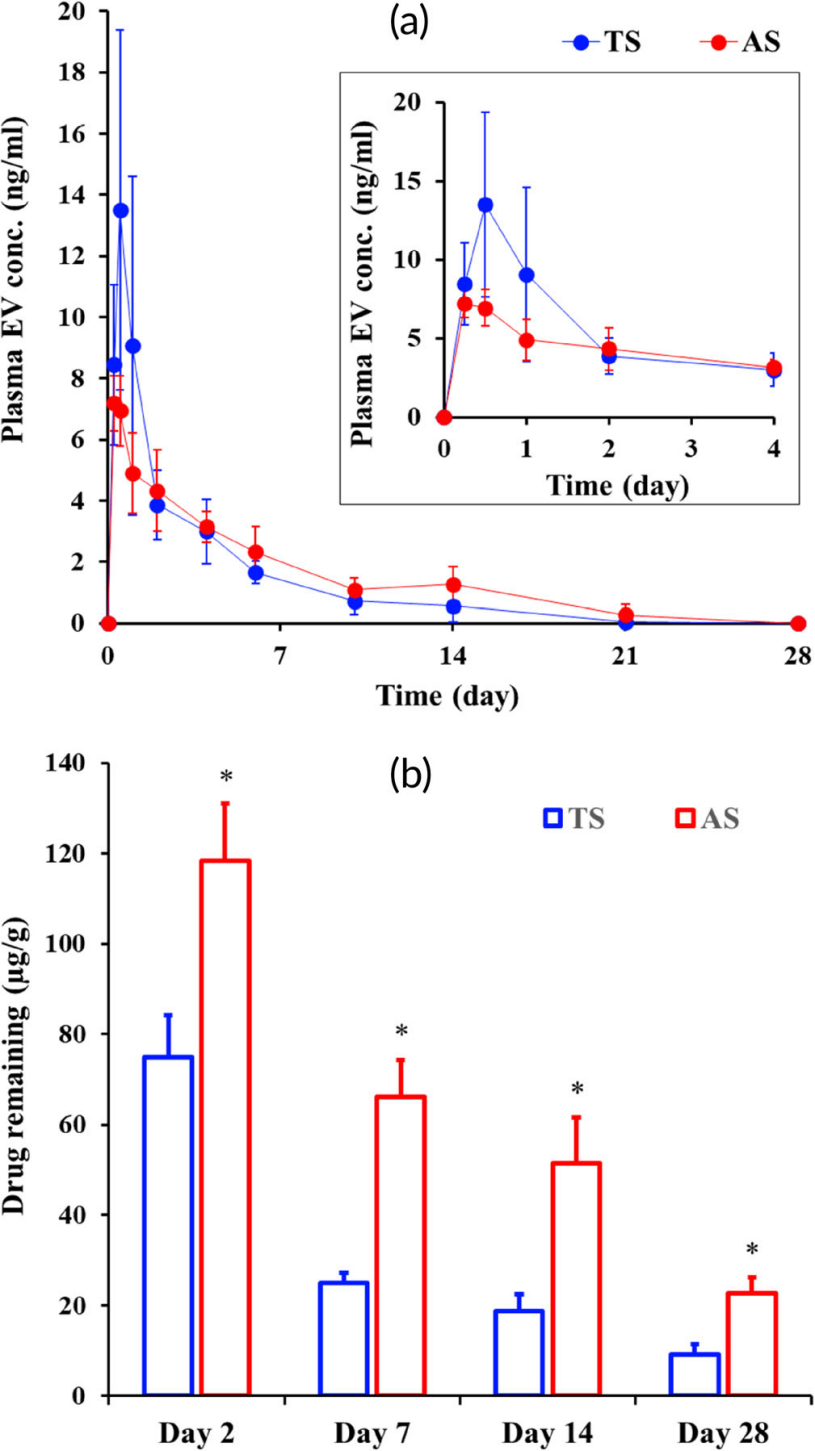
(a) Plasma concentration–time profiles of EV following IM injection (1.44 mg/kg as EV) of EV‐P TS and AS in rats. Data represent mean ± SD (*n* = 5). (b) Amount of EV‐P remaining in femoral tissue following IM injection (1.44 mg/kg as EV) of EV‐P TS and AS in rats. Data represent mean ± SD (*n* = 6) and the statistical analysis (*p < 0.05) was performed using the Student's *t*‐test. AS, aqueous suspension; EV‐P, entecavir 3‐palmitate; IM, intramuscular; TS, tricaprylin‐based drug crystalline suspension.

The in vivo plasma concentration–time profile of EV following a single IM injection of EV‐P suspensions (1.44 mg/kg as EV) in rats is shown in Figure 2a. The relevant pharmacokinetic parameters are listed in Table 3. Previously, we revealed that EV‐P molecules exposed to tissue or bloodstream were rapidly converted to the parent compound (EV) mainly by carboxylesterase.^30^ IM injection of both systems caused a rapid increase in the plasma EV level of the rats, reaching maximum drug concentration (*C*_max_) approximately 1 day following IM injection (*T*_max_ of TS and AS was 0.6 and 0.4 days, respectively). Sufficient blood supply in the muscle might have facilitated the rapid dissolution of the water‐insoluble lipidic prodrug and subsequent absorption into the bloodstream with both TS and AS. Actually, the human muscle blood flow under resting condition is estimated to approximately 11.6 mL/100 g/min and 9.6 mL/100 g/min in deltoid and gluteus tissues, respectively.^47^ Moreover, injection of foreign materials, such as drug or polymeric particles has been reported to induce muscle injuries at the injection site and promote recruitment of large quantities of interstitial fluid at injured sites.^6^ Thus, adequate infiltration of interstitial fluid into the administration site provides suitable conditions for the rapid dissolution of EV‐P particles. Although the *T*_max_ (Table 3) and in vitro dissolution rate (Figure 1h) of both formulations were comparable, there was a significant difference in the peak levels (*C*_max_) of AS and TS. *C*_max_ value obtained from TS treatment group was 13.9 ng/mL, which was approximately 1.8‐fold higher than that (7.5 ng/mL) in the AS‐treated group (Table 3). Nevertheless, *C*_max_ value of EV in plasma obtained with IM TS and AS was supposed to be tolerable, as EV showed minimal acute toxicity with oral doses up to 200 mg/kg in mice and rats.^48^ After reaching *C*_max_, there was a sharp decrease in the plasma EV levels in both suspensions comparably, decreasing to less than a third and two‐third of the *C*_max_ of TS (3.9 ng/mL) and AS (4.3 ng/mL)‐treated groups, respectively, after 2 days (Figure 2a). Afterward, the EV concentration in plasma gradually decreased over 3 weeks, and was detected below LOQ (BLOQ) of EV after 3 and 4 weeks post‐dosing of TS or AS, respectively. The elimination half‐life at terminal phase (*T*_1/2β_) was also quite different between the TS (6.1 days) and AS (9.5 days) formulations, with a longer elimination *T*_1/2_ obtained in the AS treatment group. AUC is a key pharmacokinetic parameter representing total drug exposure over time.^49^ AUC_(0–28days)_ obtained from IM injection of TS (35.3 ng∙day/mL) or AS (38.4 ng∙day/mL) was comparable, indicating that TS and AS provided equivalent extent of absorption, in spite of different absorption and elimination rates.

**TABLE 3:** Pharmacokinetic parameters of EV in plasma following IM administration of EV‐P TS and AS in rats (1.44 mg/kg as EV).

Parameters	TS	AS
AUC_(0–28days)_ (ng·day/mL)	35.31 ± 4.31	38.42 ± 4.83
*C*_max_ (ng/mL)	13.92 ± 4.35*	7.54 ± 0.41
*T*_max_ (day)	0.60 ± 0.22	0.40 ± 0.14
*T*_1/2α_ ^a^ (day)	1.00 ± 0.26	2.53 ± 1.41*
*T*_1/2β_ ^b^ (day)	6.08 ± 2.65	9.48 ± 2.89*

Abbreviations: AS, aqueous suspension; EV‐P, entecavir 3‐palmitate; IM, intramuscular; TS, tricaprylin‐based drug crystalline suspension.

^a^ Time required for the plasma concentration of EVs to decrease to half of the *C*_max_.

^b^ Time required for half of the EVs in plasma to be removed during the terminal elimination phase. The elimination phase ranged from 4 days after dosing to the end at the final points.

* Data are expressed as mean ± SD (*n* = 5) and statistical analysis (*p* < 0.05) was performed using the Student's *t*‐test.

### 2.5 Drug remaining at injection site following IM injection of EV‐P suspensions

The drug remaining in the femoral tissue following IM injection of both suspensions was evaluated as a function of time to further compare the difference in in vivo behavior TS and AS (Figure 2b). HPLC analysis showed that only EV‐P was detected, and the level of EV was BLOQ in the tissue homogenates. When considering the rapid conversion of the EV‐P molecule into a parent compound by esterase in tissue and/or blood,^30^ most injected drug particles remain in the solid state, with only a small quantity of EV‐P in the dissolved state in the femoral tissue.

As shown in Figure 2b, there was a marked difference in the amount of drug remaining at the injection site following TS or AS injection of the same doses. Overall, the level of EV‐P in the femoral tissue was gradually decreased, with more rapid decline in the TS treatment group than in the AS treatment group. Regarding the TS‐treated group, the amount of drug remaining in the femoral tissue after 2, 7, 14, and 28 days of treatment was about 75, 25, 19, and 9 μg/g, respectively, representing approximately 52.2, 20.5, 17.1, and 4.8 (w/w%) of the initial dose. On the other hand, when AS was IM injected, the amount of EV‐P in the local tissue after 2, 7, 14, and 28 days IM injection of the AS formulation was estimated to 118, 66, 51, and 23 μg/g, respectively, corresponding to 80.8, 57.8, 48.1, and 26.3 (w/w%) of the initial dose. This rapid disappearance of drug in the femoral tissue of rats injected with the TS was highly correlated with the high initial exposure of EV in plasma (Figure 2a). In contrast, the relatively slower drug disappearance following injection with AS could be attributed to extended *T*_1/2_ (*p* < 0.05) compared to TS injection (Table 3).

### 2.6 Tracking of IM injected oily and aqueous vehicles using micro‐CT

**FIGURE 3:**
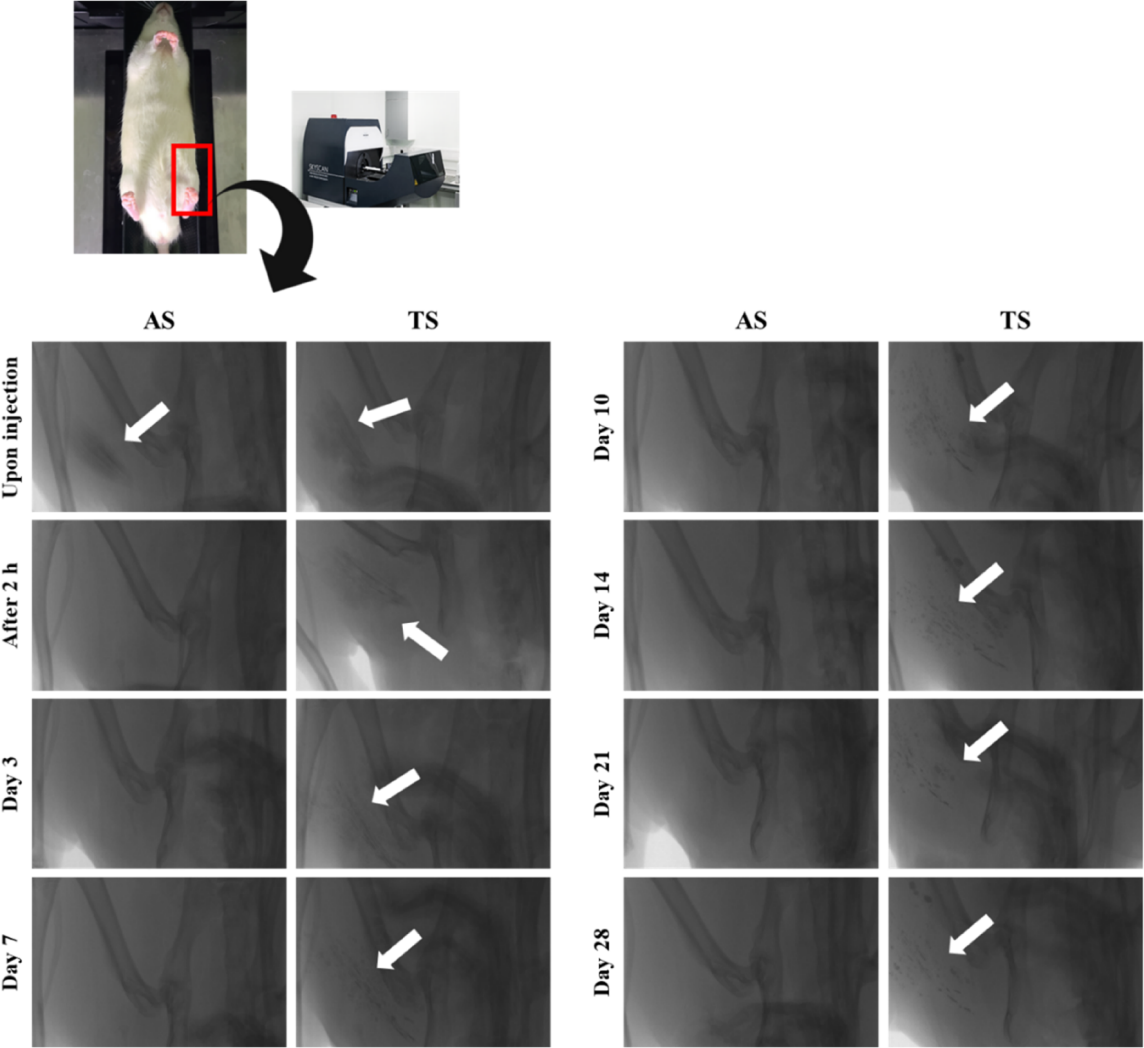
Visualization of iodinated contrast‐admixed oily and aqueous vehicle by micro CT, following IM injection in femoral tissue in rats. White arrows indicate oily vehicle remained at injection site. CT, computed tomography; IM, intramuscular.

To understand the different systemic and local pharmacokinetic profiles between TS and AS, the remaining of aqueous and oily vehicle at injection site were firstly evaluated by micro‐CT. CT is a convenient, noninvasive, and high‐resolution technology for repeated preclinical imaging of animals.^50^ As the vehicle itself is difficult to track with CT, about 30 μL of iodinated contrast agent was admixed to TS and AS (70 μL), to visualize oily and aqueous vehicles in vivo. In both formulations, dark spots originated from the iodinated contrast agents spread over the muscle fibrils were observed, after IM administration (Figure 3). In the case of AS, the signal originated from hydrophilic iomeprol was not further detected 2 h post‐injection, denoting that the iomeprol‐admixed aqueous vehicle was rapidly absorbed and distributed into the bloodstream, with high miscibility with the interstitial fluid and blood. In contrast, although the detection intensity was tended to be decreased as time elapsed, Lipiodol®‐admixed oily vehicle was still observed after 4 weeks (Figure 3). The high affinity of oily vehicle for muscle and fat components at injected tissues provided an extended retention time of the oily vehicle. This extended remaining of oily vehicle was similar to previous findings that the elimination *T*_1/2_ of castor oil, fractionated coconut oil, isopropyl myristate, and sesame oil was approximately 20, 14, 20, and 23 days, respectively, after SC or IM injection in pigs.^51^ Moreover, oil depot composed of sesame oil and benzyl alcohol (9:1, v/v) was not visible after 1 week in magnetic resonance imaging, but was still observed in histopathological evaluation even 4 weeks after IM injection.^26^ Thus, we assumed that tricaprylin would remain at the injection site along with EV‐P particles, and might affect the in vivo behavior of drug particles such as drug spreading and aggregation, dissolution process of EV‐P particles, and even biological response to the drug crystals at injection site.

### 2.7 Drug particles aggregation at injection site following IM injection

**FIGURE 4:**
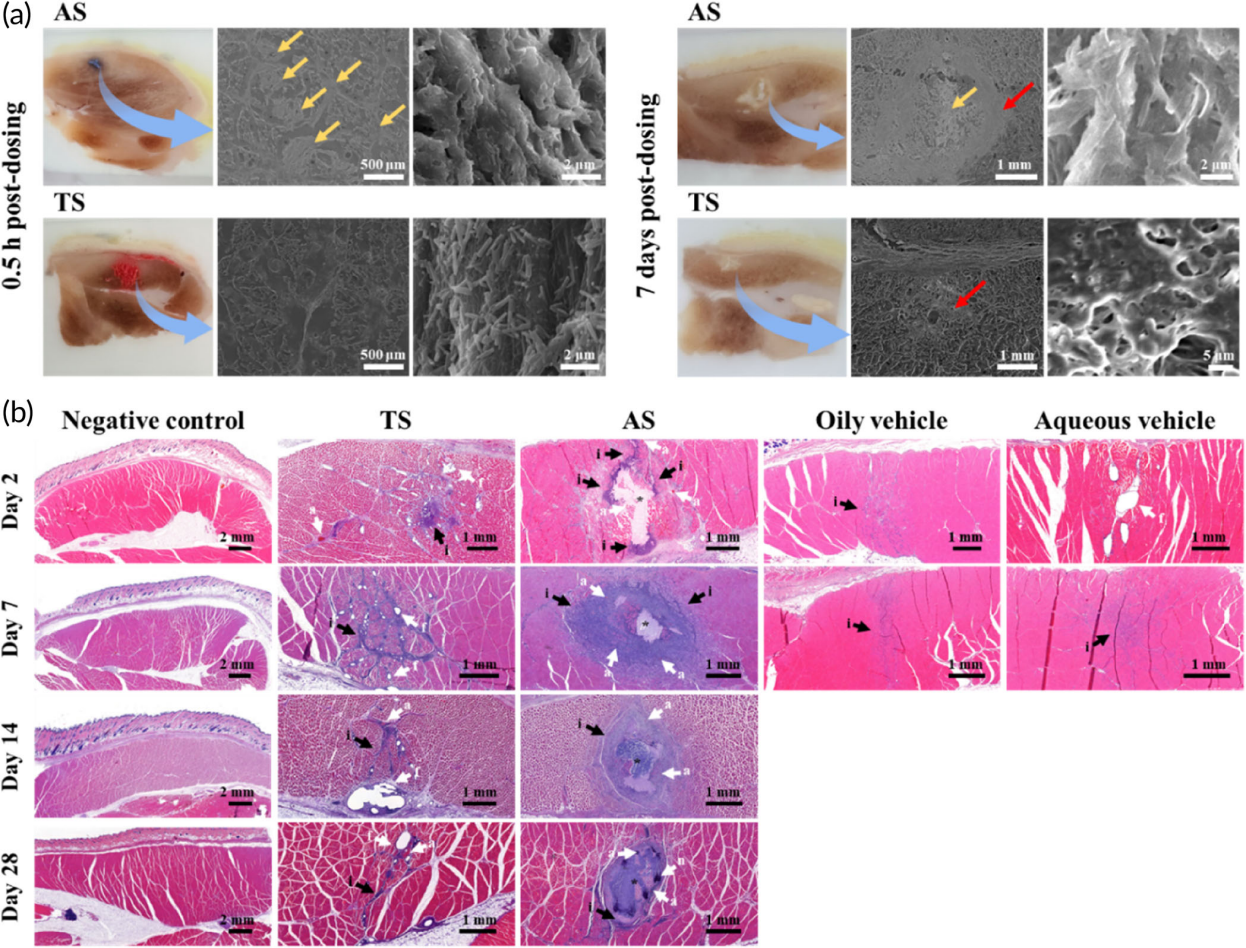
Macroscopic, microscopic, and electron‐microscopic observation of rat femoral tissues following IM injection of TS and AS. (a) Macroscopic (left panel) and microscopic (middle and right panels) images of rat femoral tissues stained by 0.1% (w/v) Sudan III (OS) and 0.01% (w/v) trypan blue (AS), respectively. (b) Histological observation of the H&E‐stained rat femoral tissues following IM injection of negative control (normal saline), vehicles (oily vehicle or aqueous vehicle), EV‐P OS, and AS. Yellow arrows and red arrows in (a) indicate drug aggregates and local inflammatory lesion at injection site, respectively. Photomicrographs of HE‐stained cross‐sections (b) show progressive foreign body reactions such as necrosis, macrophage infiltration, fibrosis, and angiogenesis following IM injection of EV‐P suspension. The depots formed by the injection of EV‐P AS are indicated by (*). Black arrows indicate granulocyte and/or macrophage infiltration (i), necrotic muscle fibers (n), and infiltrated adipocytes (f). Additional noteworthy features, such as active capillaries and angiogenesis (a), are marked with white arrows. AS, aqueous suspension; EV‐P, entecavir 3‐palmitate; H&E, hematoxylin and eosin; IM, intramuscular; TS, tricaprylin‐based drug crystalline suspension.

Next, the spreading and aggregation behaviors of EV‐P particles suspended in aqueous or oily vehicles at injected site was evaluated using an electron microscope. To exactly locate the injected site, 0.1% (w/v) of Trypan blue and Sudan III dye were admixed with TS and AS, respectively. Afterward, the injected tissues were fixed, cryo‐sectioned, and scrutinized using scanning electron microscopy (SEM). Following AS injection, white or pale white‐colored agglomeration of drug particles were observed white or at injection site after 30 min (Figure 4a, left panels). In SEM observation (Figure 4a, middle and right panels), drug agglomerates possessed rough surface, with diameter between 350 and 500 μm. The rapid absorption of the aqueous suspending vehicle at injection site caused drastic increase in surface free energy, promoting the aggregation of drug particles. Conversely, there was no recognizable aggregation of the drug particle observed at TS injection site. Actually, EV‐P particles in TS formula was observed to preserve own particular shape and diameter <3 μm and were distributed at injected tissue in SEM observation. With extended residual at injected site, the lipophilic oily vehicle (tricaprylin) might be adsorbed onto the surface of the hydrophobic drug particles, counteracting the increase in the surface free energy of particles, and thus preserving the suspended state of EV‐P particles at the injection site.

This difference in distribution and aggregation behavior between TS and AS instigated morphological difference at the injection site even 7 days post‐dosing (Figure 4a). In SEM observation, the drug cluster formed by AS injection was enclosed with a dense matrix with thickness over 0.7 mm, forming extensive inflammatory region over 6 mm^2^. Conversely, a rather narrow local inflammatory region (<2 mm^2^) was observed with TS, with no noticeable drug aggregation or depots. Through SEM observation, we revealed that the difference in the nature of the delivery vehicle markedly affects the aggregation behavior of drug particles at the injection site and the inflammatory response.

### 2.8 Histopathological observation following IM injection of EV‐P TS and AS

The femoral tissues injected with normal saline, vehicles, TS, or AS were further observed histologically using H&E stained femoral tissues (Figure 4b), to compare the local inflammatory response at the injection site and further correlate the difference in pharmacokinetic profiles of TS and AS suspensions. Injection of normal saline did not cause infiltration of inflammatory cells or tissue responses in the surrounding tissues during the experimental period (Figure 4b). On the other hand, injection of oily and aqueous vehicles caused mild infiltration of lymphocytes and granulocytes at 2 days post‐dosing. The inflammatory cell response caused by vehicle injection was almost recovered within 7 days (Figure 4b). In contrast, noticeable inflammatory lesions were observed in femoral tissues injected with both EV‐P suspensions. The tissue damage and extravasation of blood caused by EV‐P particles injection might trigger an immediate rush of inflammatory‐mediating cells to the injection site. However, the degree and/or aspect of inflammation was markedly different between TS and AS as previous observed in SEM observation (Figure 4a). On the 2nd day of injection, mild granulocyte infiltration was observed in the TS‐treated group. In contrast, muscle tissue necrosis was observed in the AS‐treated group, along with granulocyte infiltration. Seven days after dosing, the infiltration of inflammatory cells and the inflammatory response of the surrounding tissues tended to be severe in both TS and AS treatment groups 7 days post‐dosing. Particularly, the injection of AS caused the formation of a globular drug aggregate‐containing depot at the injection site, with severe desmoplastic reaction, forming fibroblastic bands around the drug depot with a macrophage rim. This granulation behavior is similar to the SEM observation that drug clusters are enclosed in the envelop (Figure 4a). After activation of the macrophages upon adherence to the drug particle, the fibrosis (i.e., fibrous encapsulation) process enclosed the drug crystals with interfacial foreign‐body reactions,^52^, ^53^ isolating the drug crystals suspended in aqueous vehicle from the local tissue environment. Being unable to phagocytose the EV‐P drug aggregates over 500 μm due to its increased particle size, macrophages and proteins such as derived from extravasated blood, such as albumin and fibrinogen, would become nonspecifically adsorbed to the surface of the drug clusters, forming macrophage layer around the drug aggregates. This granulomatous inflammatory reaction against drug particles is consistent with previous reports.^18^, ^54^, ^55^ In our previous study, the SC injection of EV‐P AS (6.3 μm, 30–150 mg/kg) caused inflammatory responses in a dose‐dependent manner, which is characterized by the drug aggregates covered with thickened fibroblastic bands, extensive infiltration of inflammatory cells, necrosis, and angiogenesis around the injection site.^54^ The aggregation of drug particles at injection site, and subsequent fibrous isolation delayed the drug dissolution and systemic absorption of the lipidic prodrug. It was also reported that the IM injection of paliperidone palmitate nanocrystals suspended aqueous vehicle caused foreign body granulomas, displaying a central, almost cell‐free depot, which was entirely surrounded by a dense inflammatory rim at 48 h after injection.^30^ The capsule principally consisted of epithelioid macrophages, T cells, vascular endothelial cells, fibroblasts, and granulocytes.^30^ After 4 weeks of AS injections, a mono‐focal depot with dense macrophage infiltration was still present, with chronic inflammation (Figure 4b). In contrast, no distinct secondary depot or fibrous capsule was observed in TS treatment tissues, despite positive staining of macrophages in the perimysium space of the muscle. The infiltration of granule leukocytes ceased; however, the infiltration of lymphocytes, a chronic inflammatory cell, was predominant after 2 weeks (Figure 4b). After 4 weeks, inflammatory lesions, macrophages, and inflammatory cells were barely observed in the TS injection site, with no indications of chronic inflammation.

The histological differences between TS and AS depending on the type of delivery vehicle could be attributed to several reasons. First, the type of vehicle might affect the spreading area and dispersion speed of drug particles at the injection site, with different absorption rate of vehicle at injection site. It could affect the distribution and aggregation behavior of drug particles at the injection site as observed by SEM (Figure 4a), with different inflammatory responses; the infiltration density of inflammatory cells and thickness of depots surrounding fibroblastic band were thicker with the large drug cluster formed by AS injection, compared to TS injection (Figure 4b). This tendency in the local inflammatory reaction is correspondent to previous report that the infiltration density of macrophages and width of the envelop surrounding drug‐containing depots tended to be thinner with smaller particle sizes; the thickness of the fibrous band obtained with 800 nm EV‐P particles was only one‐fifth of that of 22 μm‐sized drug particles after 4 weeks.^6^ Second, oily vehicle might affect the recognition and signaling pathways between inflammatory cells at the injection site, with extended retention at injection site as observed by micro‐CT (Figure 3). Although, the oily vehicle was mostly lost at the injection site within 7 days in micro‐CT observation, a small fraction of oily vehicle might be adsorbed onto the surface of the lipophilic drug particles, which may continuously impede drug recognition by host immune cells and subsequent immune response. Moreover, tricaprylin, a type of MCT, can suppress inflammatory response at the injection site, thereby alleviating inflammatory response following TS injection. In a previous study, murine macrophages (RAW 264.7) incubated with MCT showed markedly suppressed inflammatory cytokine secretion in lipopolysaccharide‐induced conditions in vitro, downregulating the production of IL‐6 and TNF‐α secretion.^12^ Treatment of murine macrophages with MCT promoted the secretion of anti‐inflammatory cytokines, including IL‐10.^29^

**FIGURE 5:**
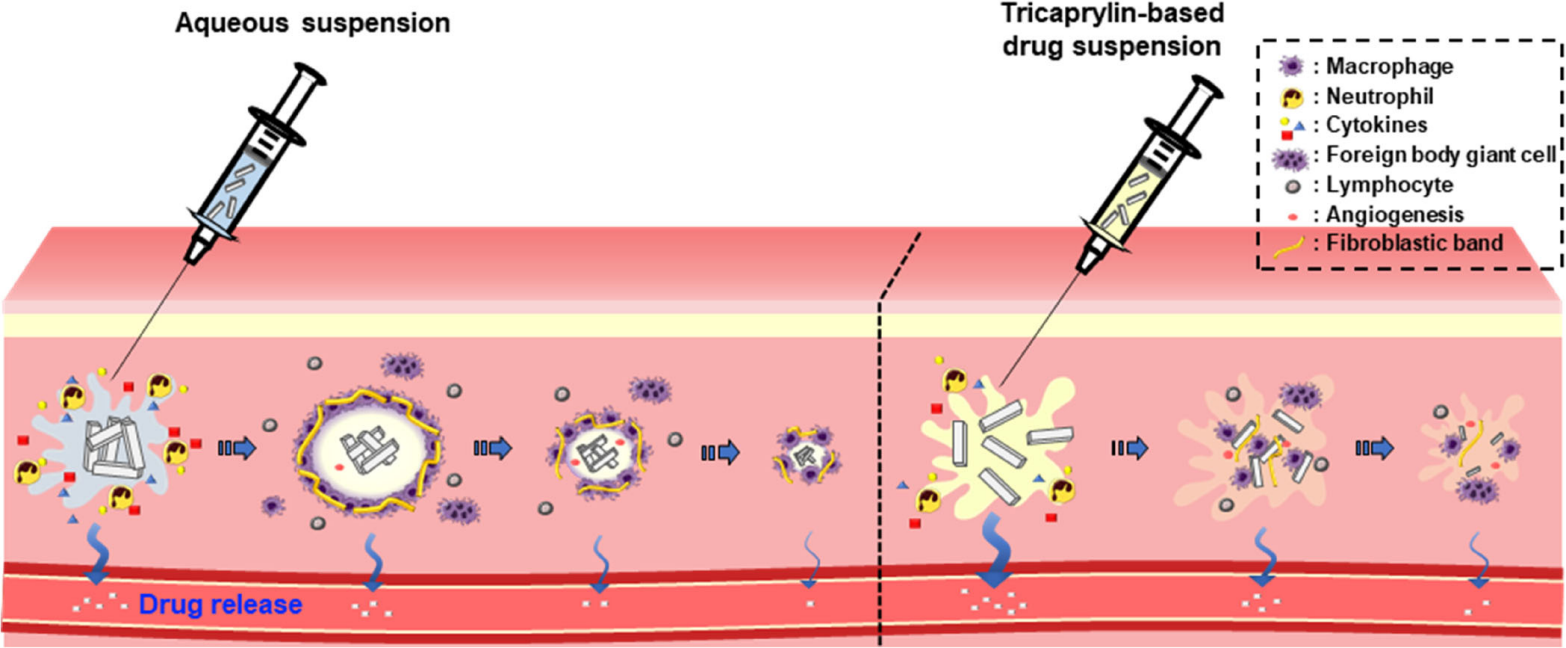
Schematic illustration of the different drug particle aggregation and corresponding local inflammatory responses, and dissolution at injection site, depending on the delivery vehicle.

These differences in remaining period of delivery vehicle, the aggregation behavior of drug particles, and corresponding local inflammatory responses at injection site were considered to be highly interconnected with the pharmacokinetic data. In the case of AS, drug aggregates‐containing depot formation and subsequent fibrous granulation increased the amount of drug remaining at the injection site, thus decreasing drug dissolution rate and subsequent drug exposure into the bloodstream, and extending the elimination *T*_1/2_. As established Noyes–Whitney equation: *dM*/*dt* = *k*⋅*S*⋅Cs, where *dM*/*dt* is the dissolution rate, *k* is the rate constant, *S* is the surface area of the drug particle, and Cs is the drug solubility, the drug particle aggregation at injection site diminished the surface area of the drug particles, and thus retarded the dissolution rate of AS system. Moreover, a dense inflammatory envelope, predominantly consisting of macrophages and fibrous matrix, surrounded the drug cluster, causing decline in dissolution and absorption rate (Figure 5). In contrast, the injection of TS did not cause formation of noticeable drug depots, facilitating drug liberation at the injection site (Figure 5). Additionally, oily vehicle remaining at the injection site could attribute to faster dissolution with higher solubility (30 μg/mL) than aqueous media (pH 7.4, 1.1 μg/mL). Actually, IM injection of EV‐P TS contributed to greater initial exposure of the parent compound, resulting in a 1.55‐fold higher AUC value for 2 days post‐injection compared with that of AS (TS, 15.91 ± 6.31; AS, 10.25 ± 2.06 ng·day/mL, data not shown). This effect of local inflammatory response on the pharmacokinetic pattern is supported by previous findings that pretreatment and intermittent dosing of clodronate, an anti‐inflammatory agent, significantly suppressed injection site response to prodrug particles by depleting blood‐derived macrophages at the injection site.^18^ The reduced inflammatory response contributed to high exposure to paliperidone, providing approximately 2.2 times higher AUC after 1 day post‐dosing, and 1.5 times higher *C*_max_, compared to IM injection with no treatment.^18^

### 2.9 Semi‐quantitative and quantitative evaluations of local tolerability of EV‐P suspensions

Tolerability at the injection site is one of the major concerns in designing drug suspension‐based LA injectable systems along with large administration volumes, drug residues at injection site, lowering the patient compliance.^34^ The bolus injection of a significant quantity of the drug dose in a solid state occasionally causes inflammatory reactions and injection‐site reactions, such as erythema, pruritus, pain, inflammation, rash, induration, itching, and edema.^56^ Thus, the local tolerability of TS and AS at the injection site was evaluated according to the ISO 10993‐6 guidelines “Tests for local effects after implantation”.

**FIGURE 6:**
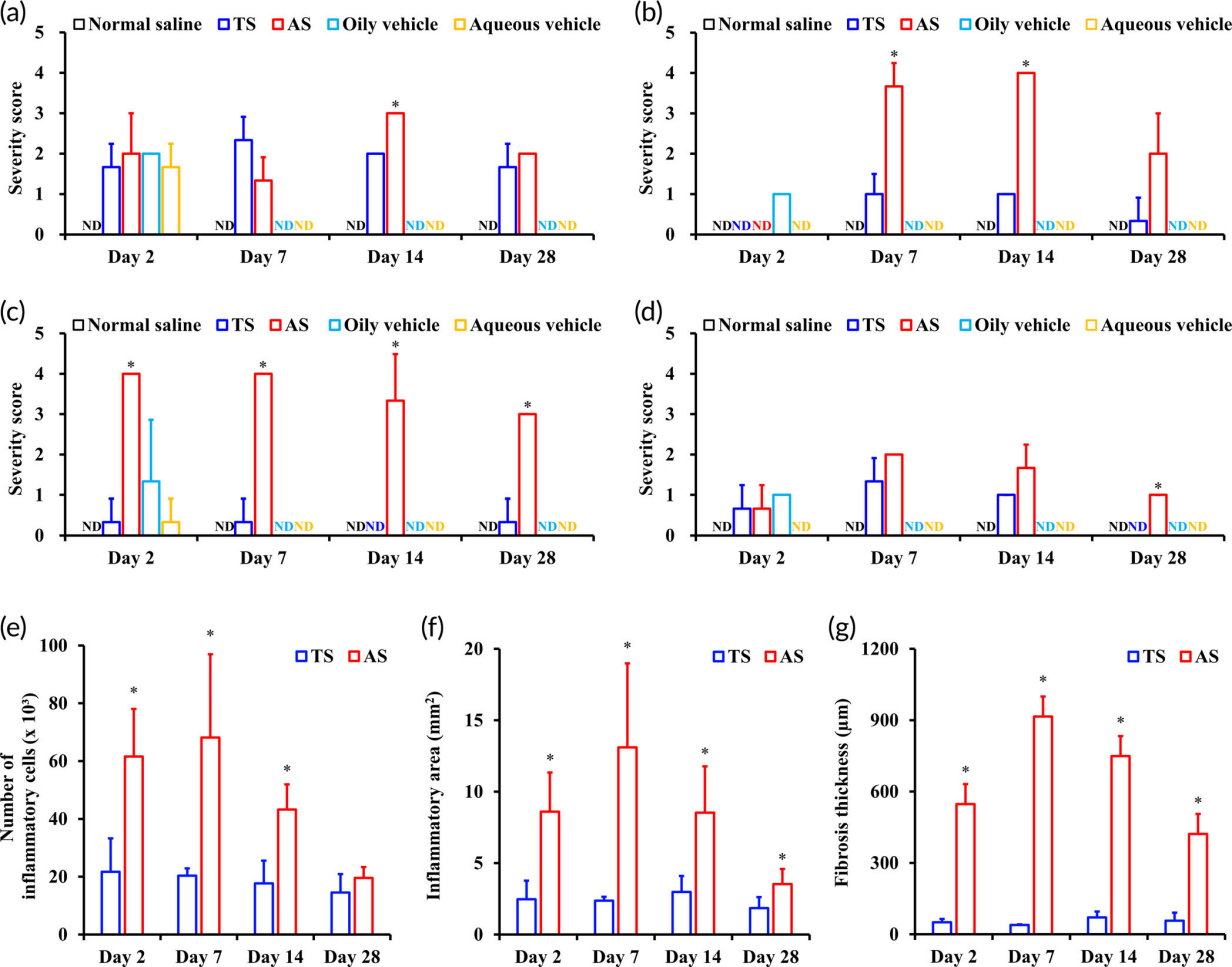
Semi‐quantitative and quantitative evaluation of inflammation severity and biological response in femoral tissue following IM injection of TS and AS. Semi‐quantitative evaluation of the severity for (a) macrophage infiltration, (b) fibrosis, (c) necrosis, and (d) neovascularization following IM administration of negative control (normal saline), vehicles (oily vehicle or aqueous vehicle), EV‐P TS, and AS. Temporal evolution of (e) numbers of inflammatory cells, (f) inflammatory area (mm^2^), and (g) fibrosis thickness (μm), following injection of EV‐P microparticles. The severity for (a) macrophage infiltration, (b) fibrosis, (c) necrosis, and (d) neovascularization, was scored from 0 to 4 by pathologist, in accordance to ISO10993‐6 guideline. Quantitative histopathological data including the (e) numbers of inflammatory cells, (f) inflammatory area, and (g) fibrosis thickness, were acquired using QuPath software by calibrating and analyzing the microscopic images of H&E‐stained sections. Significant difference compared to TS (*p < 0.05). Data represent mean ± SD (*n* = 4). AS, aqueous suspension; EV‐P, entecavir 3‐palmitate; H&E, hematoxylin and eosin; IM, intramuscular; TS, tricaprylin‐based drug crystalline suspension.

Single injection of normal saline or vehicles caused mild inflammation, but most inflammatory responses at injection site were recovered after 7 days, with severity score of 0 in aspect of infiltration of macrophages, fibrosis, necrosis, and angiogenesis (Figure 6). In contrast, the IM injection of both suspensions induced infiltration of macrophages at 2 days with a severity score of 2, and a similar severity was maintained for over 4 weeks (Figure 6a). The presence of drug particles caused moderate to marked infiltration of mononuclear cells, mainly activated macrophages. However, interestingly, there were significant differences between TS and AS in the local severity of fibrosis in the femoral tissue (Figure 6b,c), despite the comparable infiltration of macrophages at the injection site. Macrophages are the primary cells involved in biomaterial‐mediated fibrosis, which modulates inflammation and fibroblasts.^57^ Macrophages and fibroblasts communicate via juxtracrine and paracrine signaling, and the M2 macrophage‐derived signals directly influence fibroblasts to express genes and secrete proteins to promote the deposition of extracellular matrix.^57^ As shown in Figure 4a, EV‐P AS caused considerable fibrosis at the injection site, forming a fibroblastic band with newly formed blood vessels around the depot (severity score of 3.6 and 4.0 on the 7th and 14th days post‐dosing, respectively, Figure 6b). In contrast, marked depots were not observed at the TS‐injected site, with minor fibrosis response (severity score of 1 and 1 on the 7th and 14th days post‐dosing, respectively, Figure 6b). This difference indicated that tricaprylin remaining at the injection site might interfere or even suppress the signaling pathways or spatiotemporal crosstalk between macrophages and fibroblasts, with its own anti‐inflammatory effect. The degree of necrosis in muscle tissue was also markedly severe with AS injection, with severity scores of 4.0, 4.0, and 3.0, after 2, 7, and 28 days post‐dosing, respectively (Figure 6c). The severity score of the TS‐injected group was less than 0.33, throughout the experimental period. In both AS and TS‐treated groups, neovascularization peaked at 7 days post‐dosing (severity scores of AS and TS, 2.0 and 1.3, respectively, Figure 6d) and gradually decreased with time. After 4 weeks, there was almost no infiltration of inflammatory cells and neovascularization in the TS‐treated group.

The degree of inflammation at the injection site was further quantitatively evaluated based on the inflammatory area at the injection site (mm^2^), the number of total inflammatory cells in the lesion, and the thickness of fibrous capsules in H&E‐stained tissues. The total number of inflammatory cells, such as macrophages, neutrophils, and monocytes, along with the inflammatory lesions in tissues, was determined using the QuPath® program. Darville et al. revealed that the depot infiltrating mononuclear cells were mainly CD68^+^ macrophages from vascular origins, through immunohistochemistry evaluation.^18^ In the present study, compared with OS‐injected site, infiltration of inflammatory cells in AS‐injected site was considerably higher at 2, 7, and 14 days. The number of inflammatory cells in the AS‐treated group was determined to 62, 68, and 48 × 10^3^, at 2, 7, and 14 days post‐dosing, respectively, whereas that of the AS treatment group was 22, 20, and 18 × 10^3^, respectively (Figure 6e). On day 28, the number of inflammatory cells decreased below 20 × 10^3^ in both suspensions. Furthermore, there were marked difference in the inflammatory area. The inflammatory area of TS treatment site was approximately 2, 2, and 3 mm^2^ after 2, 7, and 14 days post‐dosing, which was less than a‐third of that of the AS‐treated group (Figure 6f). As indicated by histological observations (Figure 4b), AS‐injected sites had thicker fibrotic capsules, and the mean thickness of the fibroblastic band after 2, 7, 14, and 28 days was 547, 915, 748, and 421 μm, respectively, whereas that of the TS‐injected sites was less than 70 μm at every time point (Figure 6g). Based on the above semi‐quantitative and quantitative evaluations, we collectively concluded that TS could be an alternative to alleviate local inflammation compared to AS in parenteral EV‐P delivery, and the type of delivery vehicle has considerably influenced on the pharmacokinetics and local tolerability of injectable suspension.

## 3 MATERIALS AND METHODS

### 3.1 Materials

The monohydrate form of EVs was purchased from Hanseo Chem Co., Ltd. (Pyeongtaek, Korea). The lipid prodrug of EV, EV‐P powder, was synthesized, isolated, and purified in our lab, according to previously reported method.^30^ Castor oil, cotton seed oil, peanut oil, sesame oil, soybean oil, tricaprylin, triolein, oleic acid, polysorbate 20, polyethylene glycol 4000 (PEG 4000), Sudan III, 10% (v/v) neutral buffered formalin, sucrose, and sodium carboxymethyl cellulose (Na. CMC) were purchased from Sigma Chemical Co. (St. Louis, MO, USA). Labrafac PG (propylene glycol dicaprylocaprate) was a kind gift from Gattefosse (Saint‐Priest, France). Lipiodol® (ethiodized oil) and Iomeron® (iomeprol) were purchased from Guerbet (Villepinte, France) and Bracco (Milan, Italy), respectively. Zoletil was obtained from Virbac (Nice, France). HPLC‐grade acetonitrile, ethyl alcohol, and 2‐propanol were purchased from J.T. Baker (Phillipsburg, NJ, USA). Trypan blue and optimal cutting temperature (OCT) compound were obtained from Gibco™ (New York, USA) and Sakura Finetek (Torrance, USA), respectively. Other chemical reagents were of analytical grade and used without further refinement.

### 3.2 Dispersibility and solubility of EV‐P particles in oily vehicles

Pulverized EV‐P powder (5 mg as EV) was added to injectable oil vehicles (1 mL) in an Eppendorf tube. The mixture was then vortexed for 10 min using a multi‐vortex (Vortex Genie 2, Scientific Industries, Chicago, IL, USA) and then shaken for 3 h at 25°C in a shaking incubator (model BF‐60SIRL, Bio Free, Seoul, Korea). Then, the dispersibility of EV‐P particles in different oily vehicles was visually evaluated. To determine the equilibrium solubility of EV‐P in the oil vehicle, each sample stored over 3 days was centrifuged at 13,000 rpm for 10 min, and the supernatant was appropriately diluted with acetonitrile or isopropanol. The EV‐P concentration in the sample was analyzed using Waters HPLC system (Model 515 pump, Model 486 UV‐VIS detector, and Model 717 plus autosampler, Waters, Milford, MA, USA) equipped with a reversed‐phase C18 column (4.6 × 150 mm, 5 μm, Shiseido). The mobile phase for EV‐P analysis consisted of 2‐propanol and deionized distilled water at a volume ratio of 6:4. The flow rate of the mobile phase and detection wavelength were 1.0 mL/min and 253 nm, respectively, and the elution time of EV‐P was 5.3 min. The calibration curve was linear in the range of 1–100 μg/mL and the limit of detection (LOD) and limit of quantitation (LOQ) values were calculated as 0.5 μg/mL and 1.5 μg/mL, respectively.

### 3.3 Preparation of injectable EV‐P suspensions

#### 3.3.1 Preparation of TS of EV‐P

The EV‐P‐loaded TS was fabricated by pulverizing drug power into fine particles in tricaprylin (oily vehicle) using probe ultrasonicator (Model Vibra Cell, Sonics & Materials, Inc., Newtown, CT, USA).^58^ EV‐P powder (10 mg as EV) was added to tricaprylin (2 mL) and pre‐wetted for 5 min using a multi‐vortex at room temperature. The coarse suspension was placed in a scintillation vial embedded in an ice bath to prevent heating or solvent evaporation during sonication. Thereafter, the coarse suspension was sonicated using a probe ultrasonicator with a 7 mm diameter probe at 25% amplitude for 5 min, shut off for 5 min, and then reoperated at 25% amplitude for 5 min. The prepared TS was placed in a scintillation vial and stored under ambient conditions for further experiments.

#### 3.3.2 Preparation of AS of EV‐P

The AS of EV‐P was prepared by the anti‐solvent crystallization technique, following a previously described procedure with slight modification.^30^ Briefly, EV‐P powder (80 mg) was dissolved in ethanol (10 mL). A phosphate‐buffer solution (15 mM, pH 7.0) containing 0.5% (w/v) polysorbate 20 and 1.0% (w/v) PEG 4000 as stabilizer was prepared. The drug solution was then added dropwise to 30 mL of an aqueous vehicle. The organic solvent in the mixture was evaporated at a gas purging rate of 20 L/min under a stirring rate of 100 rpm. The drug concentration was adjusted to 5 mg/mL as EV (same as OS) by removing the appropriate volume of vehicle after settling the EV‐P microcrystals by centrifugation at 3500 *g* (Model Combi 508, Hanil Science, Seoul, Korea).

### 3.4 Morphological and physicochemical characterizations of EV‐P TS and AS

#### 3.4.1 Morphology of EV‐P crystals in TS and AS

Morphological features of EV‐P raw materials and drug crystals dispersed in tricaprylin or aqueous vehicle were assessed by SEM (Model JSM‐6510, JEOL, Tokyo, Japan) and TEM (Tecnai F20 G2, FEI, Hillsboro, OR, USA), respectively. For SEM observation, approximately 2 mg of EV‐P powder was loaded onto an aluminum stub using double‐sided carbon tape. The sample was then sputter‐coated using an automatic sputter coater (Model 108AUTO, Cressington, UK) at 15 mA. Micrographs of the coated samples were observed and photographed at an acceleration voltage of 15 kV. For TEM observation of EV‐P crystals suspended in AS or TS, each suspension was loaded dropwise onto the copper grid and gently blown up for 20 min to lessen the aqueous or oily vehicles. Both samples were then fixed on a sample holder and observed at an accelerating voltage of 80 kV.

#### 3.4.2 Particle size and zeta potential of EV‐P TS and AS

The crystal size of EV‐P suspended in tricaprylin or aqueous vehicle was determined using Mastersizer MS 2000 (Malvern Instruments Ltd., UK) equipped with Hydro 2000 S automatic dispersion unit.^59^, ^60^ After background alignment with each dispersion medium, samples were loaded into the dispersion unit to obtain obscuration in the range of 10%–15%. The refractive indices of the oil vehicle and aqueous vehicle were adjusted to 1.45 and 1.33, respectively. Five runs were performed for each measurement, and size distributions by volume were calculated by applying the Mie theory. The *d*_50_ was the median value defined above as the diameter at which half of the population was below this value. Similarly, 90% of the distribution was below the *d*_90_ value and 10 percent of the population was below the *d*_10_ value. The homogeneity of the microcrystals suspended in the vehicle was estimated by determining the SPAN value, which was calculated by dividing the difference between *d*_90_ and *d*_10_ by *d*_50_.^61^ The zeta potential of EV‐P crystals suspended in AS was determined using a Zetasizer Nano‐ZS (Malvern Instrument, Worcestershire, UK) at 25°C.^62^, ^63^ Each sample (100 μL) was diluted 10‐times in each vehicle and loaded into the capillary cell, and 20 runs were performed for each measurement.

#### 3.4.3 Drug content in TS and AS

The content of EV‐P suspended or dissolved in TS or AS was determined by HPLC analysis. To determine the total amount of EV‐P in the suspensions, samples (200 μL) were diluted with absolute ethanol (3.8 mL), and vortexed vigorously to obtain a clear solution. The drug solution was diluted two‐fold with the mobile phase and analyzed by HPLC. To determine the amount of EV‐P dissolved in the vehicle, each sample (1 mL) was centrifuged at 13,000 rpm for 10 min, and the supernatant was diluted two‐fold with the mobile phase and analyzed by HPLC. The quantity of EV‐P suspended in the aqueous or oily vehicles was estimated by subtracting the amount of EV‐P dissolved from the total amount of EV‐P in the suspensions.

#### 3.4.4 DSC analysis

The thermal behavior of EV‐P particles dispersed in TS or AS was examined using DSC (Model DSC 50, Shimadzu, Japan). Prior to DSC analysis, AS and TS samples were centrifuged at 13,000 rpm for 10 min to sediment EV‐P particles. The settled drug microcrystals were then collected and dried by spreading thinly on a stainless plate and stored in a desiccator (OH‐3S, AS ONE Inc., Tokyo, Japan) at room temperature. Approximately 2 mg of the dried samples were enclosed in a Tzero™ pan and lid, and then exposed to heating temperatures ranging from 40°C to 200°C at a heating rate of 10°C/min.

#### 3.4.5 X‐ray diffraction (XRD) analysis

The diffraction patterns of EV‐P particles dispersed in TS or AS was examined using an X‐ray diffractometer (Ultima IV, Rigaku Corporation, USA) equipped CuKα radiation (*λ* = 1.54 Å, 40 kV and 40 mA). EV‐P particles were collected using centrifugation process and desiccated at room temperature for 24 h. The diffraction pattern of dried samples was then analyzed at 25°C in the range from 10° to 40° at 0.02° steps.

#### 3.4.6 Viscosity of EV‐P TS and AS

The apparent viscosities of both EV‐P suspensions were determined using a rotational shear rheometer (ARES‐G2, TA instrument Ltd., DE, USA) equipped with a Peltier stage and parallel plate.^64^ Approximately 2 g of drug suspension was placed on the lower plate (40 mm diameter) with a geometric gap of 1 mm. The temperature of the conditioning step was set to 25°C, and then the viscosity of the samples was determined at shear rates (s^−1^) ranging from 10^−1^ to 0.9 × 10^2^. During the measurement, the % tolerance at each point was set to 5.0%.

### 3.5 In vitro dissolution profile of EV‐P from suspensions

The in vitro release profiles of EV‐P particles from TS and AS were comparatively evaluated using dialysis bag diffusion model.^65^, ^66^, ^67^ Approximately 1 mL of each suspension containing 9.3 mg of EV‐P was placed inside Float‐A‐Lyzer® device equipped with a dialysis membrane (100 kDa MWCO, Spectrum Lab, CA, USA). Then, the drug‐loaded device was immersed in 200 mL of dissolution medium maintained at 37 ± 0.5°C and agitated at 300 rpm. The dissolution media consisted of a mixture of isopropyl alcohol and distilled water at a volume ratio of 50:50 to guarantee the sink condition and miscibility of the oily vehicle to the media. At predetermined intervals, 1 mL of dissolution medium was withdrawn and centrifuged at 13,000 rpm for 10 min. The supernatant (500 μL) was then diluted two‐fold with methanol, and the EV‐P concentration in the aliquot was determined by HPLC as described above. The equivalent volume of fresh pre‐warmed dissolution medium was then replenished to the beaker to maintain the volume of the dissolution medium.

### 3.6 In vivo pharmacokinetic evaluation in rats

The plasma concentration–time profile and the amount of EV remaining at the injection site following IM injection of TS or AS were evaluated in rats with approval from the Institutional Animal Care and Use Committee (IACUC) of Dankook University (approval number: DKU‐19‐033). Eight‐week‐old male Sprague–Dawley rats (250 ± 20 g) were acquired from Samtako (Kyungki‐do, Korea) and were housed in a controlled animal room (23 ± 1°C and 50 ± 5% relative humidity) under a 12‐h light–dark cycle.

For the in vivo pharmacokinetic study, the rats were divided randomly into two groups (*n* = 5 per group) after an acclimatization period of 3 days: TS and AS treatment groups. Uniform suspensions (TS or AS) were administered intramuscularly to the femoral tissue of the hind leg at a dose of 1.44 mg/kg (as EV) using a 31 G insulin syringe. Afterwards, the rats were allowed to move freely in the cage with free access to water and standardized chow. At predetermined intervals, approximately 0.2 mL of blood sample was collected from the submandibular vein using a 26 G heparinized syringe (heparin at 20 IU). Within 1 h of collection, plasma was separated by centrifugation at 4000 rpm for 10 min at 4°C and stored in a deep freezer at −80°C. After thawing at ambient temperature, the level of EV in plasma was determined by HPLC mass spectroscopy (MS/MS) as previously described.^13^ Pharmacokinetic parameters, such as area under the plasma concentration versus time curve from zero to 28 days (AUC_0–28days_), maximum plasma concentration (*C*_max_), time to peak maximum plasma concentration (*T*_max_), and half‐life (*T*_1/2_) were calculated using pharmacokinetic analysis program (WinNonlin® version 5.2, Pharsight Co., Mountain View, CA, USA).

The amount of drug remaining at the drug injection site following IM injection was comparatively evaluated in rats. The rats were randomly divided into two groups (TS and AS; *n* = 12 per group) and the drug suspensions were administered to both femoral tissue (1.44 mg/kg as EV per site) in the same manner as in the pharmacokinetic study. At predetermined time points (2, 7, 14, and 28 days post‐treatment), three rats from each treatment were euthanized at each time point by chloroform inhalation, followed by cervical dislocation. Both hind legs were carefully excised using surgical equipment (*n* = 6 per point) to remove excessive skin, muscle, and bone. The accurately weighed tissue was then soaked in absolute ethanol (5 mL) and vigorously vortexed for 24 h to extract the remaining EV‐P and EV. Samples were centrifuged at 13,000 rpm for 10 min, and the supernatant was diluted two‐fold with the mobile phase of EV‐P and EV. The levels of EV‐P and EV in the aliquot were determined by HPLC, as described above.

### 3.7 Tracking of IM injected oily and aqueous vehicles using micro‐CT

All experiments involving animals were approved by the IACUC of Dankook University (approval number: DKU‐19‐033, date of approval: October 8, 2019). The retention behavior of each vehicle in the two suspensions at the injection site was estimated by micro‐CT scan (Skyscan 1176, Bruker Corporation, Billerica, MA, USA) of the femoral tissue of experimental rats.^68^, ^69^ To enable the traceability of both vehicles in CT observation, 300 μL of Lipiodol®, a lipophilic iodinated contrast, was added to 700 μL of TS, whereas the hydrophilic contrast agent (Iomeron®, 300 μL) was added to AS (700 μL). After an acclimatization period of 3 days, 100 μL of each formulation was injected intramuscularly to the femoral tissue of the experimental rats (Samtako, Kyungki‐do, Korea). At predetermined times, rats were anesthetized by chloroform inhalation and SC Zoletil (100 μL) injection and were placed in 6 cm bed under supine position. Injection site was then scanned using a micro‐CT imaging system at a pixel size of 18 μm, X‐ray source voltage of 80 kV, and source current of 300 μA.

### 3.8 SEM observation of EV‐P particles injected femoral tissues

Spreading and aggregation behaviors of drug particles at the injection site, depending on delivery vehicles, were evaluated in rats after approval from the IACUC of Dankook University (approval number: DKU‐19‐033). Eight‐week‐old male Sprague–Dawley rats (250 ± 20 g) acquired from Samtako (Kyungki‐do, Korea) were housed in a controlled animal room (23 ± 1°C and 50 ± 5% relative humidity) under a 12‐h light–dark cycle. For visual observation, AS and TS were colored by addition of 0.01% (w/v) trypan blue (blue color) and 0.1% (w/v) Sudan III (red color), respectively. Stained suspensions (TS or AS) were administered intramuscularly to the femoral tissue of the hind leg at a dose of 1.44 mg/kg (as EV) using a 31 G insulin syringe. At predetermined time points (0.5 h and 7 days post‐injection), femoral tissues were surgically isolated, and the tissues were subjected to tissue treatment; isolated femoral tissues were fixed in 10% (v/v) neutral buffered formalin for 72 h at 4°C. Fixed tissues were washed by 1× PBS and stored in 30% sucrose solution at 4°C. Dehydrated tissues were trimmed to fit into the cryomold and embedded in OCT compounds. Samples frozen at −28°C were sectioned using cryostat cryocut microtome (Model CM3050S, Leica, Wetzlar, Germany) with thickness of 25 μm. Tissue sections were then scrutinized using SEM, as described in Section 3.4.1.

### 3.9 In vivo local inflammatory responses at injected sites following IM injection

#### 3.9.1 Drug administration, tissue fixation, and H&E staining

In vivo local inflammatory responses in the injected femoral tissue following IM injection of normal saline, aqueous, or tricaprylin, AS, or TS were evaluated in male 8‐week‐old Sprague–Dawley rats (250 ± 20 g). Rats in the treatment groups were injected intramuscularly (1.44 mg/kg per site) with TS or AS into both femoral regions of the hind leg, whereas those in the control groups were injected with equal volumes of normal saline or aqueous or oily vehicles of TS and AS. At predetermined time points (2, 7, 14, and 28 days post‐dosing), femoral tissues were surgically isolated, and the tissues were subjected to tissue treatment procedures previously reported.^18^ Briefly, isolated femoral tissues were fixed in 10% (v/v) paraformaldehyde for 72 h at 40°C and subjected to routine processing for histological analysis. Next, paraffin‐embedded tissue specimens were sectioned into 10 μm thickness using a microtome (Model Leica RM2165, Wetzlar, Germany). The sections were then deparaffinized with two steps of xylene and dehydrated through descending ethanol grades (100%, 90%, 80%, and 75%). and stained with hematoxylin and eosin.

#### 3.9.2 Histological observation, semi‐quantitative, and quantitate evaluations of inflammatory responses at injection site

H&E‐stained sections of injected sites were captured using a Pannoramic 250 Flash digital microscope (P250 Flash digital microscopes) equipped with CaseViewer software (3DHISTECH, Budapest, Hungary). Histological parameters evaluated at the injection sites included the shape or area of the depot and the degree of inflammatory infiltration, such as polymorphonuclear cells, macrophages, lymphocytes, plasma cells, giant cells, necrosis, angiogenesis, and fibrosis. The severity of local inflammation was assessed semi‐quantitatively by pathologist (Chemon, Yongin‐si, Gyeonggi‐do, Korea), by scoring the degree of infiltration of macrophage, necrosis, neovascularization, and fibrosis, according to the criteria of ISO 10993‐6 guideline “Tests for local effects after implantation”.^70^ The presence of macrophages was scored as follows: 0, none; 1, rare [1–5 per high powered (×400) field)]; 2, 5–10 per field; 3, heavy infiltrate; and 4, packed. The severity of necrosis was scored as follows: 0, none; 1, minimal; 2, mild; 3, moderate; and 4, severe. Neovascularization was scored as follows: 0, none; 1, minimal capillary proliferation (1–3 buds); 2, groups of 4–7 capillaries with fibroblastic structures; 3, broad band of capillaries with supporting structure; and 4, extensive band of capillaries with fibroblastic structure. The degree of fibrosis was scored as follows: 0; 1, narrow band; 2, moderately thick band; 3, thick band; and 4, extensive band.

The inflammatory area, the number of inflammatory cells, and the thickness of the fibroblastic band were further evaluated quantitatively for the slices with the widest inflammatory lesion for each individual subject using QuPath® open‐source image analysis software (Queen's University, Belfast, UK).^71^, ^72^, ^73^ The inflammatory area was determined by contouring the injected depot area using the wand tool, and the area was automatically calculated based on the scale of the images. Cell counting was performed automatically after the loaded images were fine‐tuned. The pixel size was adjusted depending on the brightness and contrast of the image, and the nucleus parameter was set from 5 to 400 μm^2^. The thickness of fibrosis around the depot was directly determined by measuring the length of the pale fibrous tissues on the upper, lower, left, and right sides of the fibrous rim, using the shape statistics tool of the program.

### 3.10 Statistical analysis

The differences in pharmacokinetic parameters between TS and AS were statistically analyzed using Student's *t*‐test. Histological semi‐quantitative and quantitative data were statistically analyzed using one‐way analysis of variance (ANOVA), followed by post‐hoc analysis for multiple comparisons (SPSS Software 17, SPSS Inc., Chicago, IL, USA). Statistical significance was set at *p* < 0.05.

## 4 CONCLUSIONS

Novel EV‐P crystalline suspension employing tricaprylin, an injectable neutral oil as delivery vehicle was engineered, and it's in vivo performance was delicately evaluated with comparison to conventional AS. Despite comparable crystal size and crystallinity, TS system provided markedly lessened inflammatory response following IM injection compared to AS, with slow disappearance of the oily vehicle at injection site. While drug clusters or secondary depots were not formed with TS, drug aggregates‐containing depot surrounded with fibroblastic bands were instigated by AS injection, with severe inflammatory response. IM injection of both TS and AS offered prolonged pharmacokinetic profile of the anti‐viral agent, over 3 weeks. From these findings, we concluded that TS system could be an alternative for prolonged delivery of the water‐insoluble drug particles including EV‐P, to alleviate inflammatory response at injection site.

## AUTHOR CONTRIBUTIONS

**Min Young Jeong:** Conceptualization (equal); data curation (equal); writing – original draft (equal). **Myoung Jin Ho:** Conceptualization (equal); data curation (equal); writing – original draft (equal). **Joon Soo Park:** Formal analysis (equal). **Hoetaek Jeong:** Formal analysis (equal). **Jin Hee Kim:** Formal analysis (equal). **Yong Jin Jang:** Formal analysis (equal). **Doe Myung Shin:** Formal analysis (equal). **In Gyu Yang:** Methodology (equal). **Hye Rim Kim:** Methodology (equal). **Woo Heon Song:** Methodology (equal). **Sangkil Lee:** Supervision (equal). **Seh Hyon Song:** Methodology (equal). **Yong Seok Choi:** Supervision (equal); writing – review and editing (equal). **Young Taek Han:** Supervision (equal). **Myung Joo Kang:** Funding acquisition (equal); methodology (equal); project administration (equal); supervision (equal).

## FUNDING INFORMATION

This research was supported by Basic Science Research Program through the National Research Foundation of Korea (NRF) funded by the Ministry of Science, ICT & Future Planning (NRF‐2022R1C1C1004561).

## CONFLICT OF INTEREST STATEMENT

The authors declare no conflicts of interest.

### PEER REVIEW

The peer review history for this article is available at https://www.webofscience.com/api/gateway/wos/peer‐review/10.1002/btm2.10649.

## Data Availability

The data that support the findings of this study are available from the corresponding author upon reasonable request.
